# The anti-asthmatic drug, montelukast, modifies the neurogenic potential in the young healthy and irradiated brain

**DOI:** 10.1038/s41419-018-0783-7

**Published:** 2018-07-10

**Authors:** Yohanna Eriksson, Martina Boström, Åsa Sandelius, Kaj Blennow, Henrik Zetterberg, Georg Kuhn, Marie Kalm

**Affiliations:** 1 Department of Pharmacology, Institute of Neuroscience and Physiology, Sahlgrenska Academy, University of Gothenburg, Gothenburg, Sweden; 2 Department of Oncology, Institute of Clinical Sciences, Sahlgrenska Academy, University of Gothenburg, Gothenburg, Sweden; 3 Department of Psychiatry and Neurochemistry, Institute of Neuroscience and Physiology, Sahlgrenska Academy, University of Gothenburg, Mölndal, Sweden; 4 Clinical Neurochemistry Laboratory, Sahlgrenska University Hospital, Gothenburg, Sweden; 5 Department of Molecular Neuroscience, UCL Institute of Neurology, Queen Square, London, UK; 6 UK Dementia Research Institute, UCL, London, UK; 7 Center for Brain Repair and Rehabilitation, Institute of Neuroscience and Physiology, Sahlgrenska Academy, University of Gothenburg, Gothenburg, Sweden; 8 Center for Stroke Research Berlin, Charité – Universitätsmedizin, Berlin, Germany

## Abstract

Brain tumors are the most common form of solid tumors in children. Due to the increasing number of survivors, it is of importance to prevent long-term treatment-induced side effects. Montelukast, a leukotriene receptor antagonist, may have the desired neuroprotective properties. The aim of the study was to determine whether montelukast could reduce adverse effects of cranial irradiation (CIR) to the young brain. Daily injections of montelukast or vehicle was given to young mice for 4 or 14 days in combination with CIR or under normal conditions. Montelukast treatment for 4 days protected against cell death with 90% more cell death in the vehicle group compared to the montelukast group 24 h after CIR. It also resulted in less microglia activation 6 h after CIR, where montelukast lowered the levels of CD68 compared to the vehicle groups. Interestingly, the animals that received montelukast for 14 days had 50% less proliferating cells in the hippocampus irrespective of receiving CIR or not. Further, the total number of neurons in the granule cell layer was altered during the sub-acute phase. The number of neurons was decreased by montelukast treatment in control animals (15%), but the opposite was seen after CIR, where montelukast treatment increased the number of neurons (15%). The results show beneficial effects by montelukast treatment after CIR in some investigated parameters during both the acute phase and with longer drug treatment. However, it also resulted in lower proliferation in the hippocampus under normal conditions, indicating that the effects of montelukast can be either beneficial or unfavorable, depending on the circumstances.

## Introduction

Children with asthma receive daily treatment and according to international guidelines the first choice of treatment is inhaled corticosteroids. The treatment is often combined with adrenergic β_2_ receptor agonists. The second choice for treatment is leukotriene receptor antagonists, for example, montelukast (Singulair®), which is approved for use in children (<12 years)^1^. It is not unusual that the first and second choices are combined. Montelukast has been considered well tolerated in children due to mild and transient side effects^2^. However, current reports show that montelukast treatment can cause psychiatric adverse drug reactions in children, such as sleep disorder, anxiety, aggressiveness, and hyperactivity with an incidence higher than 10%^3–5^. The underlying reasons for these side effects remain unknown.

Leukotrienes are normally present at low levels in the brain but increase during pathological conditions^6^. Montelukast has been described to be neuroprotective, for example, in the aging brain and after focal cerebral ischemia^7,8^. In the aging brain, increased activation of microglia is accompanied with decreased hippocampal neurogenesis and cognitive decline^7^. Daily, oral administration of montelukast to rats for 6 weeks improved these negative changes in the aging brain^7^, effects that could also be beneficial in radiotherapy-induced injury in children.

Brain tumors are the most common form of solid tumors in children^9^, with surgery, chemotherapy, and radiotherapy as the main treatment strategies^10^. All of these treatments cause late side effects, with radiotherapy having the highest severity when graded^11^. Side effects range from sleep disturbance to cognitive impairment^12^. The mechanisms of chronic radiation-induced damage involve, for example, long-term toxicity to neural cell types, including stem and progenitor cells, loss of oligodendrocytes, and inflammatory responses^13^. Cranial radiotherapy also changes the chemical milieu and affects supporting cells such as microglia^14,15^. The subgranular zone (SGZ) in the hippocampus, an area in the brain that harbor stem cells, is very sensitive to radiotherapy in both the young and adult brain, and loss of these cells may contribute to cognitive deficits^16–18^. Finding means to ameliorate radiation-induced injury is of great interest for the increasing number of long-term childhood cancer survivors.

Targeting the irradiation-induced inflammatory response is of interest to prevent negative effects on cognition and neurogenesis. Inhibiting microglia with MW-151, a selective inhibitor of proinflammatory microglial cytokine production, restored hippocampal-dependent learning, improved synaptic function, and partially protected neurogenesis after cranial irradiation (CIR) to the adult rat brain^19^. Further, it has been shown that indomethacin, a common nonsteroidal anti-inflammatory drug, has the potential to partly increase neurogenesis after CIR in adults^20^. Blocking chemokine (C–C motif) receptor 2 (Ccr2) has also prevented neuronal dysfunction and hippocampal-dependent memory dysfunction induced by irradiation toward the adult mouse brain^21^. We have previously shown that the juvenile and adult brain have different radiation-induced inflammatory responses^22^, which is of importance if using an anti-inflammatory approach to protect the brain from CIR-induced injury. In the developing brain, it has been shown that blocking the complement cascade can improve reversal learning after CIR, but without effects on neurogenesis^23^. As mentioned earlier, montelukast is a leukotriene receptor antagonist. Leukotrienes are lipid mediators of inflammation and are metabolized from arachidonic acid through the 5-lipoxygenase (5-LOX) pathway. It has been shown that minocycline, a tetracycline antibiotic that blocks the activation of 5-LOX, had positive effects on cognitive impairment and decreased apoptosis in newborn neurons (DCX^+^) following a single dose of 20 Gy irradiation to the brain of 1 month old rats^24^. Inhibiting the 5-LOX pathway to target the inflammatory response could therefore be an interesting strategy to investigate when trying to protect the normal tissue during radiotherapy. The purpose of this study was to investigate the effect of montelukast in combination with CIR to the young brain.

## Results

**Fig. 1:**
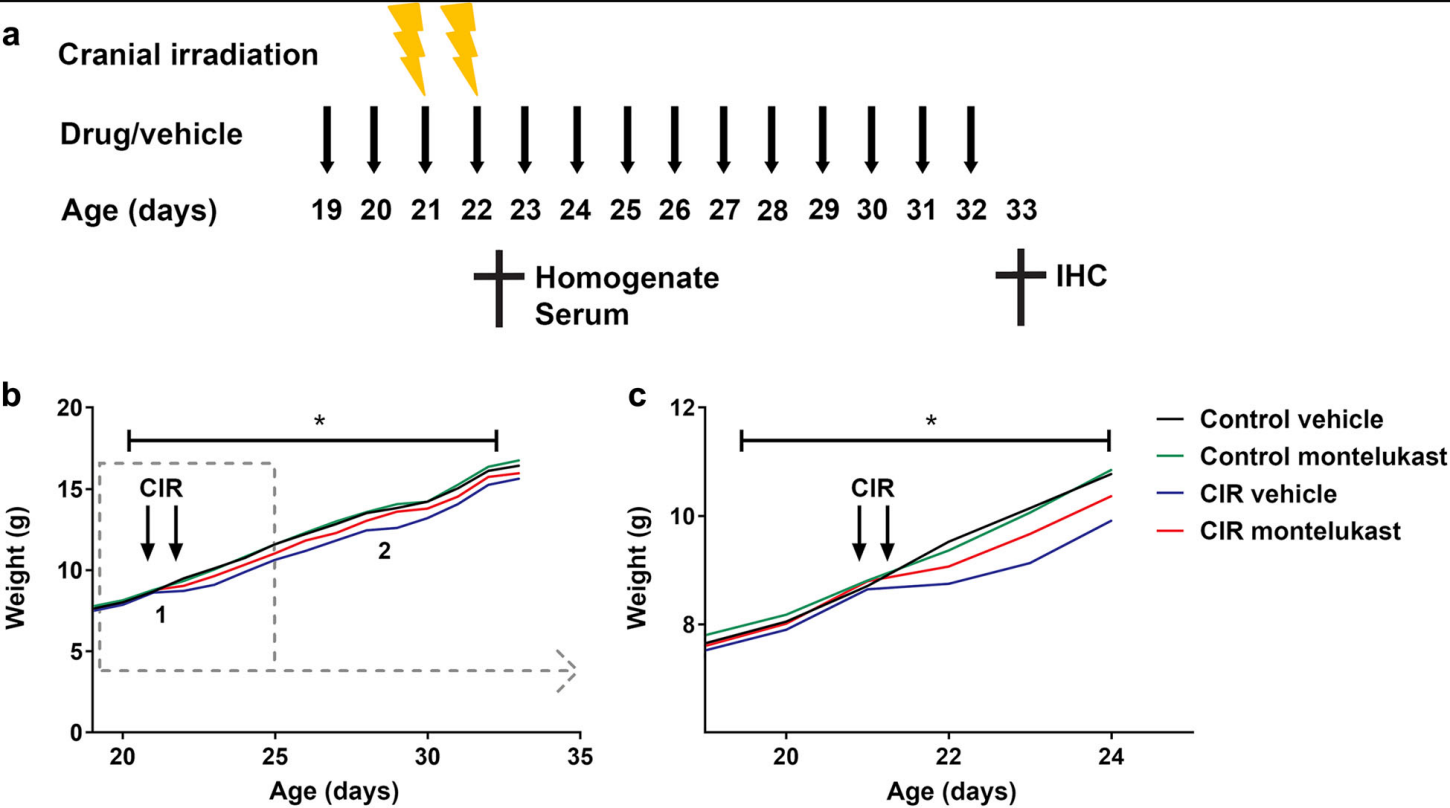
Study outline and weight gain. **a** Study outline. The weight was measured daily throughout the experiment. The entire period is shown in (**b**). A lag in weight gain was observed in the groups that received CIR (1). The montelukast groups weaned 1 day later than the vehicle groups (2). The days before and after CIR are zoomed in (**c**). CIR cranial irradiation, IHC immunohistochemistry. † = sacrifice. *n*_control vehicle_ = 10, *n*_control montelukast_ = 9, *n*_CIR vehicle_ = 6, *n*_CIR montelukast_ = 7. Statistical results from three-way ANOVA are presented in the figures, * = interaction between treatment, drug, and time. *P = 0.036

The study outline is presented in Fig. 1a. Weight gain was carefully monitored for all mice injected daily for 14 days and an interaction between treatment, drug, and time was observed (*P* = 0.036, Fig. 1b–c). CIR resulted in a significantly delayed weight gain compared to control animals and stayed below the other groups throughout the experiment. Interestingly, the montelukast group did not show the same delayed weight gain following CIR.

### Acute phase following CIR

**Fig. 2:**
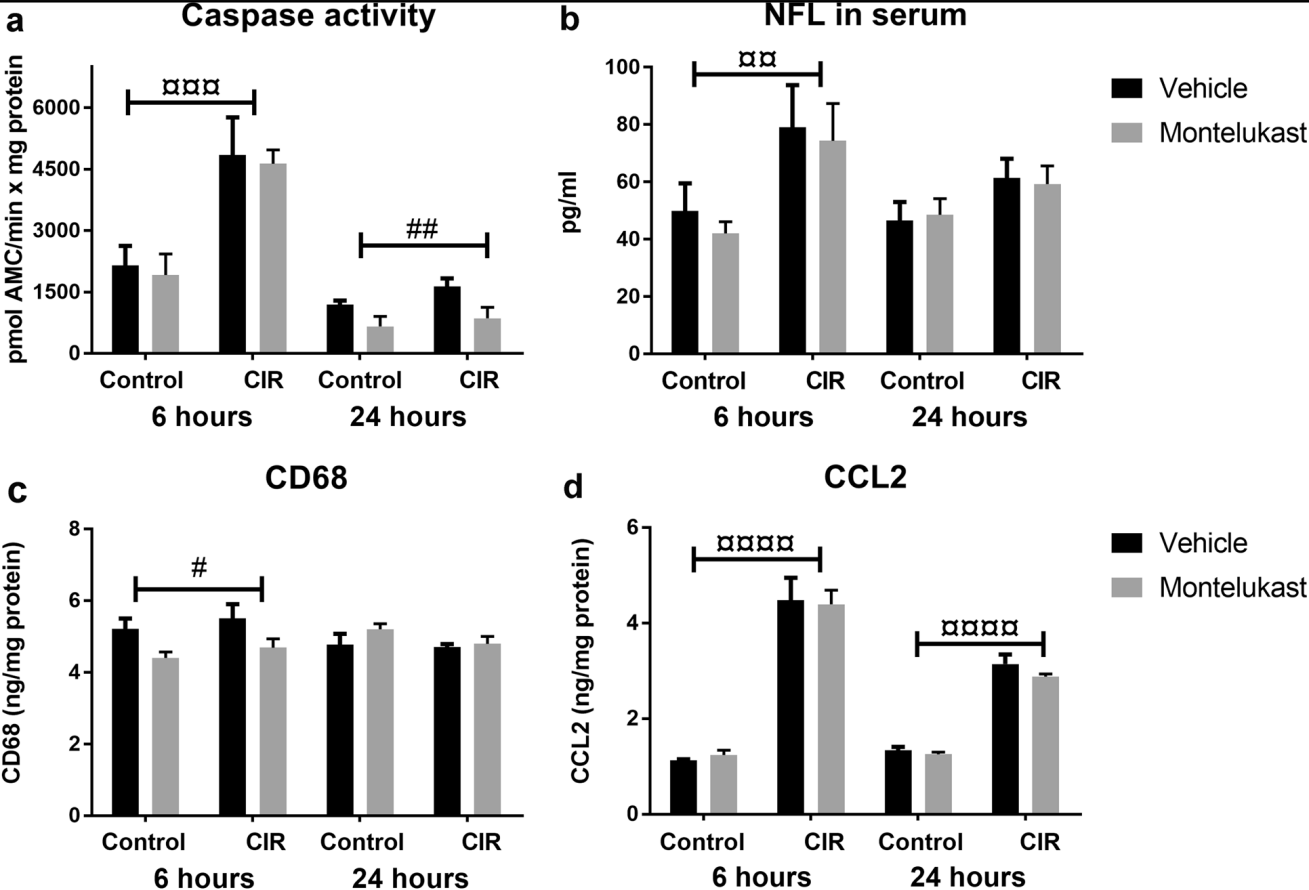
Markers of neuronal injury and inflammation were measured following CIR and montelukast treatment. **a** Levels of caspase-3/7-activity, reflecting cell death. **b** NFL in serum, reflecting neuronal injury. **c** Levels of CD68, reflecting microglial activation. **d** Levels of CCL2, reflecting the proinflammatory response. CCL2 chemokine (C–C motif) ligand 2, CD68 cluster of differentiation 68, CIR cranial irradiation, NFL neurofilament light chain. **a** *n*_control vehicle 6 h_ = 5, *n*_control montelukast 6 h_ = 5, *n*_CIR vehicle 6 h_ = 5, *n*_CIR montelukast 6 h_ = 5, *n*_control vehicle 24 h_ = 5, *n*_control montelukast 24 h_ = 4, *n*_CIR vehicle 24 h_ = 5, *n*_CIR montelukast 24 h_ = 5. **b** *n*_control vehicle 6 h_ = 9, *n*_control montelukast 6 h_ = 9, *n*_CIR vehicle 6 h_ = 5, *n*_CIR montelukast 6 h_ = 6, *n*_control vehicle 24 h_ = 7, *n*_control montelukast 24 h_ = 7, *n*_CIR vehicle 24 h_ = 5, *n*_CIR montelukast 24 h_ = 5. **c**, **d** *n* = 5 for all groups. Error bars indicate ± SEM. Statistical results from two-way ANOVA are presented in the figures. ¤ = treatment (control/CIR), # = drug (vehicle/montelukast), ^#^*P* < 0.05, ^¤¤^*P* < 0.01, ^¤¤¤^*P* < 0.001, ^¤¤¤¤^*P* < 0.0001

To investigate the acute CIR-induced injury, caspase activity in brain homogenate and neurofilament light chain (NFL) in serum was measured. Cell death (caspase-3/7-activity) increased to similar levels in both the vehicle and the montelukast group at 6 h after CIR (*P* *=* 0.0004, Fig. 2a). The relative increase after CIR was 125% in the vehicle groups (post hoc, *P* = 0.0121) and 141% in the montelukast groups (post hoc, *P* = 0.0116). Interestingly, the levels of caspase activity had decreased back to normal levels at 24 h after CIR. However, there was a drug effect at this time (*P* = 0.0078, Fig. 2a) with 90% more cell death in the CIR vehicle group compared to the CIR montelukast group (post hoc, *P* = 0.0357). The level of NFL, reflecting neuronal injury, increased in serum 6 h after CIR (*P* = 0.0055, Fig. 2b). This trend was seen in both the vehicle (post hoc, 58%, *P* = 0.1136) and the montelukast groups (post hoc, 77%, *P* = 0.0565). However, no difference was observed between the different groups at 24 h after CIR.

Cluster of differentiation 68 (CD68) and chemokine (C–C motif) ligand 2 (CCL2) were measured in brain homogenate to evaluate the inflammatory response following CIR. The level of CD68 was significantly altered 6 h after CIR (*P* = 0.0128, Fig. 2c). At this time point montelukast lowered the levels of CD68 compared to both vehicle groups (post hoc, n.s.). The levels of CCL2 increased significantly after CIR in both vehicle and montelukast groups at both time points (*P* < 0.0001, Fig. 2d). The vehicle group showed a CIR-induced increase of 298% (post hoc, *P* *<* 0.0001) and the montelukast group a CIR-induced increase of 254% (post hoc, *P* < 0.0001) 6 h after the injury.

### Sub-acute phase following CIR

**Fig. 3:**
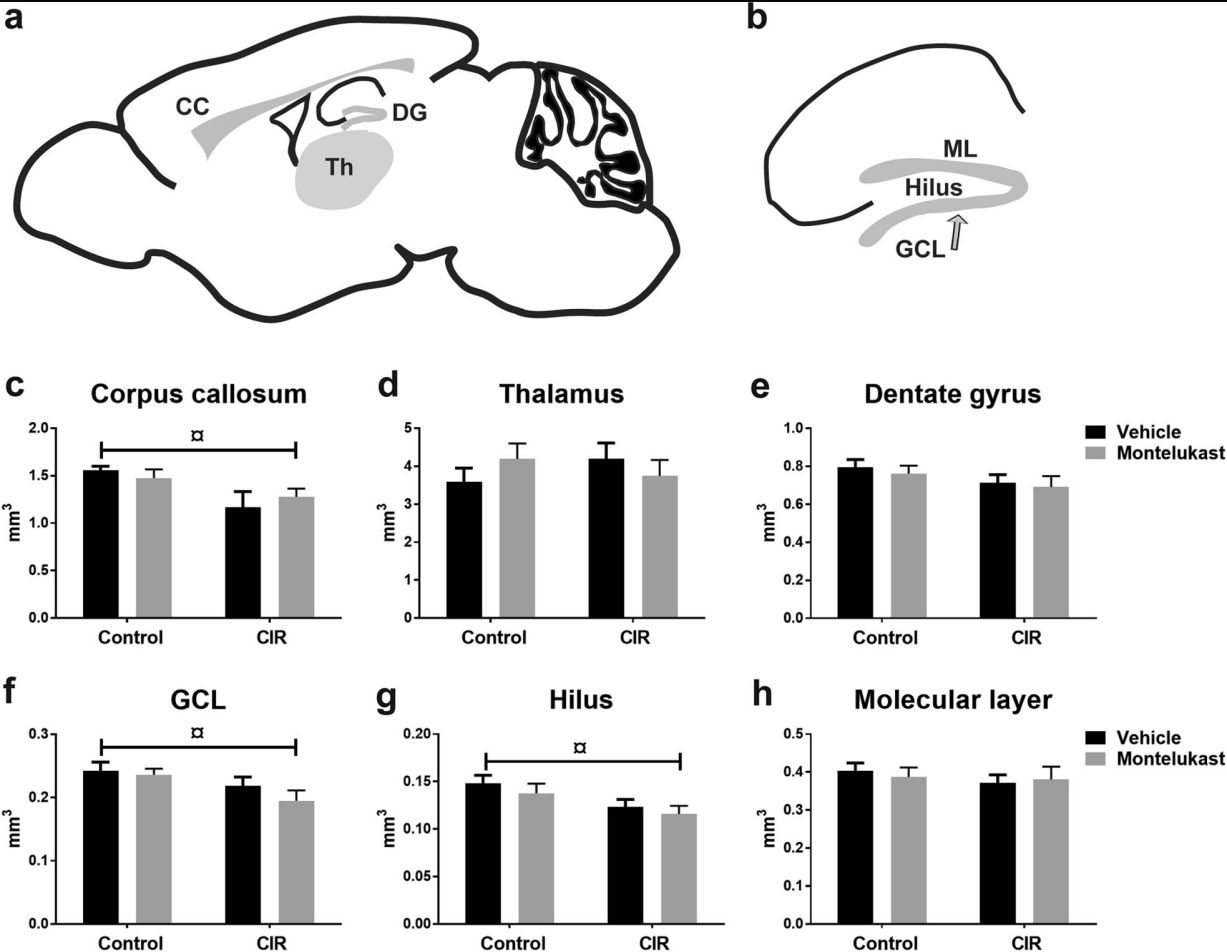
Volumetric alterations following CIR and montelukast treatment. **a** Investigated areas are highlighted in the schematic overview. The hippocampal regions are illustrated in **b**. The volumes were measured in the corpus callosum (**c**), thalamus (**d**), and hippocampal dentate gyrus (**e**). The subregions of the dentate gyrus; GCL (**f**), hilus (**g**), and molecular layer (**h**) are also presented. CC corpus callosum, CIR cranial irradiation, DG dentate gyrus, GCL granule cell layer, ML molecular layer, Th thalamus. **c** *n*_control vehicle_ = 7, *n*_control montelukast_ = 9, *n*_CIR vehicle_ = 6, *n*_CIR montelukast_ = 5. **d** *n*_control vehicle_ = 9, *n*_control montelukast_ = 9, *n*_CIR vehicle_ = 6, *n*_CIR montelukast_ = 7, **e**, **f**, **g**, **h** *n*_control vehicle_ = 8, *n*_control montelukast_ = 9, *n*_CIR vehicle_ = 6, *n*_CIR montelukast_ = 5. Error bars indicate ± SEM. Statistical results from two-way ANOVA are presented in the figures, ¤ = treatment (control/CIR), ^¤^*P* < 0.05

Twelve days after CIR, the volumes of corpus callosum, thalamus, and dentate gyrus were measured (Fig. 3a–b). The volume of corpus callosum was smaller following CIR for both vehicle and montelukast groups (*P* = 0.0115, Fig. 3c). For vehicle animals, it was 25% smaller (post hoc, *P* = 0.0334) and for the montelukast group the decrease was 13.4% (post hoc, n.s). No difference was observed in the thalamic region or the dentate gyrus (Fig. 3d–e). However, an analysis of the subregions in the dentate gyrus revealed that the volumes of the granule cell layer (GCL, *P* = 0.0228, Fig. 3f) and hilus (*P* = 0.0243, Fig. 3h) changed following CIR. The CIR vehicle group had a smaller volume compared to the control vehicle group in the GCL (9.8%, post hoc, n.s.) and the hilus (16.6%, post hoc, n.s.). The CIR montelukast group also had a smaller volume compared to the non-irradiated montelukast group in the GCL (17.4%, post hoc, n.s., *P* = 0.0827) and the hilus (16.0%, post hoc, n.s.). There was no difference in the molecular layer (ML) (Fig. 3h).

**Fig. 4:**
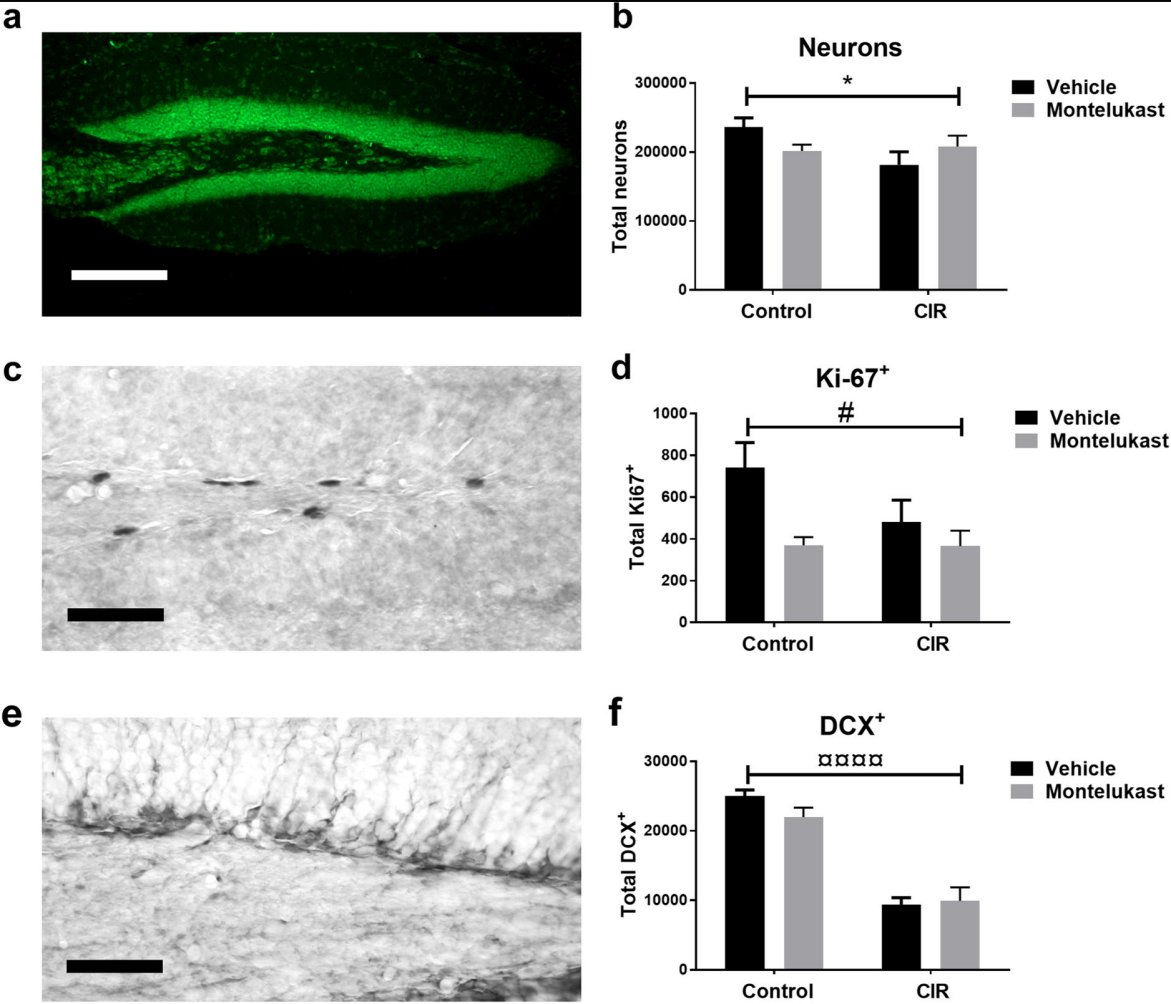
Cellular effects in the GCL following CIR and montelukast treatment. **a** A microphotograph of Neurotrace^TM^ in the dentate gyrus. **b** Neurons were quantified in the GCL where a significant interaction was observed. **c** Proliferating cells, assessed by Ki-67^+^ staining. **d** Ki-67^+^ quantification in the hippocampal neurogenic SGZ. **e** Newborn cells, measured as total numbers of DCX^+^ cells. **f** DCX^+^ quantification in the SGZ. **a** Scale bar 20 µm. **b** *n*_control vehicle_ = 8, *n*_control montelukast_ = 9, *n*_CIR vehicle_ = 6, *n*_CIR montelukast_ = 6. **c** Scale bar 50 µm. **d** *n*_control vehicle_ = 9, *n*_control montelukast_ = 8, *n*_CIR vehicle_ = 5, *n*_CIR montelukast_ = 4. **e** Scale bar 50 µm. **f** *n*_control vehicle_ = 8, *n*_control montelukast_ = 9, *n*_CIR vehicle_ = 6, *n*_CIR montelukast_ = 5. CIR cranial irradiation, DCX doublecortin, GCL granule cell layer, SGZ subgranular zone. Error bars indicate ± SEM. Statistical results from two-way ANOVA are presented in the figures, ¤ = treatment (control/CIR), # = drug (vehicle/montelukast), * = interaction between treatment and drug. *P < 0.05, ^#^*P* < 0.05, ^¤¤¤¤^*P* < 0.0001

Cellular effects in the GCL were assessed by determining the total numbers of neurons (Neurotrace^TM^), proliferating cells (Ki-67^+^) and newborn neurons (doublecortin, DCX^+^). Analysis of the total number of neurons revealed a significant interaction between treatment and drug (*P* = 0.0364, Fig. 4a–b). The vehicle CIR group had 23% less neurons in the GCL compared to vehicle controls. This was not observed in the montelukast group, where CIR did not alter the levels of neurons. Interestingly, montelukast treatment decreased the number of neurons with 15% when comparing vehicle controls and montelukast controls. Proliferation was assessed by quantifying the number of Ki-67^+^ cells (Fig. 4c–d). Montelukast decreased proliferation during normal conditions with 50% (post hoc, *P* = 0.0291), while CIR did not significantly alter the proliferation levels. The total number of newborn neurons (DCX^+^) was decreased in the GCL after CIR but not altered by montelukast (*P* < 0.0001, Fig. 4e–f). CIR treatment decreased the level of newborn neurons with 63% in the vehicle CIR group compared to vehicle controls (post hoc, *P* < 0.0001) and 55% in the montelukast CIR group compared to montelukast controls (post hoc, *P* < 0.0001).

**Fig. 5:**
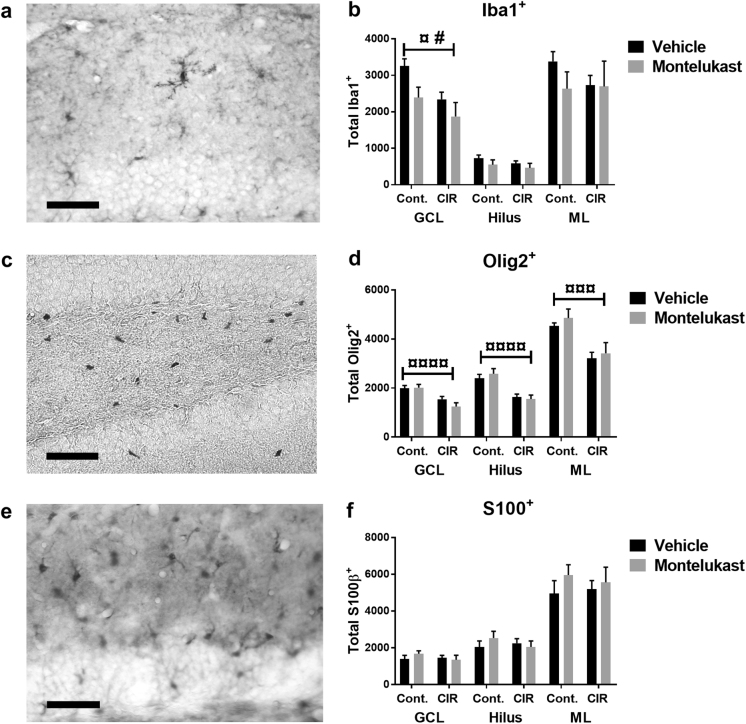
Effects on non-neuronal cells in the dentate gyrus following CIR and montelukast treatment. A microphotograph (**a**) and quantifications (**b**) of microglia (Iba1^+^) in subregions of the dentate gyrus (GCL, hilus, and ML). A microphotograph (**c**) of oligodendrocytes (Olig2^+^), and subsequent quantifications in subregions of the dentate gyrus (**d**). A microphotograph (**e**) and quantifications (**f**) of astrocytes (S100^+^) in subregions of the dentate gyrus. CIR cranial irradiation, GCL granule cell layer, Iba1 ionized calcium-binding adapter molecule 1, ML molecular layer, Olig2 oligodendrocyte transcription factor 2, S100 S100 calcium-binding protein. **a**, **c**, **e** Scale bar = 50 µm. **b** *n*_control vehicle_ = 8, *n*_control montelukast_ = 9, *n*_CIR vehicle_ = 6, *n*_CIR montelukast_ = 5. **d** *n*_control vehicle_ = 7, *n*_control montelukast_ = 8, *n*_CIR vehicle_ = 6, *n*_CIR montelukast_ = 5. **f** *n*_control vehicle_ = 10, *n*_control montelukast_ = 9, *n*_CIR vehicle_ = 6, *n*_CIR montelukast_ = 7. Statistical results from two-way ANOVA are presented in the figures, ¤ = treatment (control/CIR), # = drug (vehicle/montelukast), ^¤#^*P* < 0.05, ^¤¤¤^*P* < 0.001, ^¤¤¤¤^*P* < 0.0001

To investigate effects on non-neuronal cells in the dentate gyrus, microglia (Iba^+^), oligodendrocytes (Olig2^+^), and astrocytes (S100^+^, possibly including a subpopulation of neurons) were quantified. The number of microglia was affected by both montelukast treatment (*P* = 0.0233) and CIR (*P* = 0.015, Fig. 5a–b). However, the post hoc test only revealed a difference between the vehicle control group and the montelukast CIR group in GCL (post hoc, *P* = 0.0066). Similar trends were observed in the hilus and ML. Further, the number of oligodendrocytes decreased following CIR in GCL (*P* < 0.0001), hilus (*P* = 0.00005), and ML (*P* = 0.000243, Fig. 5c–d). No effect was observed following montelukast treatment. In the GCL, the vehicle CIR group had 23% less oligodendrocytes compared to the vehicle controls (post hoc, *P* = 0.039). For the montelukast CIR group, the relative reduction of oligodendrocytes in the GCL was 38% compared to the montelukast control group (post hoc, *P* = 0.0007). Similar changes were observed in the hilus and the ML for both vehicle and montelukast groups. The number of S100^+^ cells was not affected by montelukast or CIR treatment (Fig. 5e–f).

## Discussion

Radiotherapy is an effective treatment for brain tumors but unfortunately causes long-lasting side effects in the brain. In this study, parameters that are known to be affected by CIR were investigated to evaluate the effect of montelukast on radiation-induced injury in the developing brain. The major findings were the following: (1) Montelukast treatment resulted in reduced cell death during the acute phase after CIR. (2) The number of neurons was altered by montelukast treatment with a positive effect after CIR, but with a negative effect during normal conditions. (3) Proliferation in the hippocampal neurogenic zone decreased by montelukast treatment. These data indicate that the effects of montelukast can be either beneficial or unfavorable, depending on the physiological conditions.

Delayed weight gain has previously been observed after CIR to the developing brain^25^, but the underlying mechanism is not clear. Two possibilities, which are often seen in patients, are alterations in the hormonal balance or injury to the mucous membrane. A CIR-induced injury in the mucosa in the mouth and throat would make it painful for the mice to eat. Regarding the hormonal levels, the thyroid hormones FT3 and FT4 have been shown to be unaffected 4 months after CIR in a similar mouse model^26^. Presumably, the delayed weight gain is an effect of both hypothalamic–pituitary axis dysfunction and injury in the mucous membrane. Here, CIR caused a significantly delayed weight gain in the vehicle group but not in the montelukast group. The different reactions after CIR suggest that montelukast may have a protective effect on the physiological response to CIR, possibly due to its anti-inflammatory effects.

Acute cell death and increased levels of the neuronal injury marker, NFL, in serum are two expected findings following CIR, especially in a still developing brain^27,28^. In this study, the vehicle group exhibited more cell death compared to the montelukast group acutely following CIR. It has been demonstrated that montelukast could increase the proliferation of neuronal precursor cells in vitro through the receptors CysLT1R and GPR17^29^. However, there was no effect on cell fate or differentiation in that study. In this in vivo study, we observed the opposite. Montelukast rather had a negative effect on both proliferation and maturation under control conditions. It is possible that montelukast administration immediately negatively affected the cell proliferation, resulting in less proliferating cells at the time of CIR. This would then be reflected by less cell death. Further, it has been shown that microglia express, for example, GPR17^30,31^, and it is therefore possible that montelukast have a direct impact on microglia which would affect both the levels of CD68 and Iba1. Also, microglia themselves could affect the injury since they are known producers of reactive oxygen species and proinflammatory factors^32^, and excess of these factors can worsen brain injury. Interestingly, montelukast had a positive effect on the number of mature neurons after CIR, but not on the number of proliferating cells or newborn neurons. Hence, the survival of newborn neurons was positively affected in this scenario.

Montelukast has been tested in neurological conditions where inflammation and cognitive dysfunction is of interest. For example, montelukast acutely protects cerebral tissue in neonatal rats following ischemic brain damage^33^. These findings are in line with our results with lower levels of cell death in the montelukast group following CIR. Another study has shown beneficial effects on spatial memory and cognition in the aging brain after montelukast treatment^7^. An in vitro study showed an increased proliferation of neuronal progenitors after montelukast administration, but using higher doses it decreased proliferation^29^. This support our finding that montelukast could have a negative effect on neuronal proliferation in the intact juvenile brain.

Whether montelukast is beneficial or not seems to depend on the absence or presence of injury in the young brain. Positive results from montelukast treatment have been seen in the aging brains of rats after a dose of 10 mg/kg body weight^7^. The dose to treat asthma in children (2–5 years old) is however 4 mg/day regardless of body weight. In this study, we have examined the effects of montelukast in the young brain after CIR at the same dose as in the aging study, 10 mg/kg, but during a shorter time course compared to the clinical situation. Our aim was to investigate if this dose of montelukast could have similar protective effects after CIR, as the damage resembles the aging brain with elevated inflammation and less proliferation. Nevertheless, our findings could also contribute to explaining some alterations that montelukast induce in the young healthy brain as we have seen negative effects both acutely and after 2 weeks of daily administration of montelukast. However, more studies are needed to explore the therapeutic window for montelukast in pediatric patients. It should be emphasized that asthma itself can induce hypoxia in the brain, leading to, for example, cognitive dysfunction^34^. A future perspective could be to investigate if montelukast is a good treatment in such cases to control asthma and at the same time minimize injuries from the hypoxia for this group of children.

In summary, montelukast has negative effects on the maturation of the GCL during normal conditions, whereas during a pathological condition, such as following CIR, the effects can be protective. These findings, with the affected proliferation during normal conditions, in combination with the new profile for psychiatric adverse drug reactions, suggests that prescribing montelukast to young children should be a well thought through decision. However, more studies are needed to investigate if the negative effects are occurring also at lower dose spans and if the effect is chronic if ending the treatment with montelukast.

## Material and methods

### Animals

C57BL/6J mice with six female pups per mother were ordered from Charles River Laboratories, Germany. Animals were housed according to normal procedures at the Experimental Biomedicine animal facility (University of Gothenburg, Gothenburg, Sweden). The mice were kept on a 12-h light cycle with food and water provided *ad libitum.* The room temperature was 19–21 °C with 40–70% relative humidity. Animal experiments were approved by the Gothenburg committee of the Swedish Animal Welfare Agency (2015–72).

### Montelukast treatment

Montelukast sodium powder (Sigma, USA) was dissolved in 99.5% ethanol, diluted 1:10 with 0.9% saline (NaCl), and administered by intraperitoneal injections at a dose of 10 mg/kg. Animals (*n* = 6–10) received daily injections of montelukast or vehicle (0.9% NaCl with 10% ethanol) starting 2 days before CIR, resulting in four doses for the acute study and 14 doses for the sub-acute study (Fig. 1a).

### Cranial irradiation procedure

A linear accelerator True Beam STX (600 MU/min, 5.6 Gy/min; Radiation Oncology Systems, USA) with 6 MV nominal photon energy was used for CIR as described earlier^28^. Briefly, all animals were anesthetized on postnatal day 21 with a mixture of oxygen and isoflurane (Attane Vet, VM Pharma AB, Sweden) to immobilize the animals during the procedure. The whole brain was irradiated with a clinically relevant dose of 2 × 4 Gy with 12-h interval, using a radiation field of 2 × 2 cm, a source to skin distance of 99.5 cm, and a dose variation of ±5%. After CIR, animals were returned to their dams. Control animals were anesthetized but did not receive CIR. Typically, pediatric patients with brain tumors are treated with radiotherapy, once a day, 5 days per week, for several weeks. The treatment protocol varies depending on tumor type and other relevant factors, but often it is 50–59.4 Gy in 28–33 fractions of 1.8 Gy. This study was designed to investigate the radiation-induced injury in the normal tissue (8 Gy given in two fractions), hence a much lower dose than the tumor bed receive.

### Acute tissue preparation

Brains were quickly removed after sacrifice, put in liquid nitrogen and stored at −80 °C. Brains were homogenized by sonication in phosphate-buffered saline containing Triton X-100 (Merck KGaA, Germany), ethylenediaminetetraacetic acid (EDTA, Sigma-Aldrich, USA), and protease inhibitor cocktail (cOmplete, EDTA-free, Roche, Switzerland). Samples were then centrifuged and supernatant stored at −80 °C. Protein concentration was measured using the Pierce BCA protein Assay Kit (Thermo Scientific, USA) according to the protocol provided by the manufacturer.

### Sub-acute tissue preparation

Animals were anesthetized with sodium pentobarbital (Pentothal, Electra-box Pharma, Sweden) and transcardially perfused with 0.1 M phosphate buffer (pH 7.5) to rinse the vascular system, followed by 6% formaldehyde (pH 7.4; Histofix; Histolab Products AB, Sweden). Brains were gently removed, immersion-fixed in Histofix for 24 h and stored in a sucrose solution (30% sucrose in 0.1 M phosphate buffer, pH 7.5). The right hemisphere was cut sagittally into 25 µm sections in a series of 12, using a sliding microtome (SM2010R, Leica Microsystems, Germany), and stored at 4 °C in a cryoprotectant solution (25% ethylene glycol and 25% glycerol).

### Blood collection

Mice were anesthetized with a mixture of oxygen and isoflurane, blood was drawn from the heart with a 1 mL syringe (Omnifix®, BRAUN, Germany) and centrifuged for 5 min. Serum was collected and stored at −80 °C.

### Fluorometric assay of caspase-3-like activity

Caspase-dependent cell death was measured using a caspase activity assay. An aliquot of 20 µl tissue homogenate was added to a microplate and mixed with 80 µl extraction buffer (*n* *=* 5, duplicate samples) and analyzed as described earlier^35^. Cleavage of Ac-DEVD-AMC (for caspase-3/7-activity, Peptide Institute, Japan, cat. no.3171-v) was measured and expressed as pmol AMC released per mg protein and minute.

### Enzyme-linked immunosorbent assay

Enzyme-linked immunosorbent assays (ELISA) were used to investigate chemokine (C–C motif) ligand 2 (CCL2, MJE00, R&D Systems, USA) and cluster of differentiation 68 (mouse CD68, EKM1518, Nordic BioSite, Sweden) expression. Analyses were performed according to instructions of the manufacturers and the amount of investigated proteins measured using a SpectraMax i3x (Molecular Devices, USA).

### Serum NFL

Serum sample neurofilament light chain (NFL) concentration was determined using an in-house NFL assay on the single molecule array (Simoa) platform, which has been described in detail previously^36^. Briefly, paramagnetic carboxylated beads (Quanterix Corp, USA) were coated with a mouse anti-NFL antibody (UD1, UmanDiagnostics, Sweden) and incubated with sample and a biotinylated mouse anti-NFL antibody (UD2, UmanDiagnostics, Sweden) in a Simoa HD-1 instrument (Quanterix).

### Immunohistochemistry

Sections were treated as follows: rinsed (always in Tris-buffered saline [TBS], 50 mM Tris-HCl in 150 mM NaCl), incubated in 0.6% H_2_O_2_ in TBS for 30 min, rinsed and incubated for 30 min in a TBS block solution with 3% donkey serum (Jackson ImmunoResearch Laboratories Inc, USA), and 0.1% Triton X-100 (Merck KGaA, Germany). Sections were incubated overnight at 4 °C in block solution using the following primary antibodies: oligodendrocyte transcription factor 2 (1:500, rabbit anti-Olig2, ab109186, Abcam, UK), marker of proliferation Ki-67 (1:1000, rabbit anti-Ki-67, ab15580, Abcam, UK), S100 calcium-binding protein (1:2000, mouse anti-S100, MA5-12969, ThermoFisher Scientific, USA), rabbit anti-ionized calcium-binding adapter molecule 1 (1:1000, rabbit anti-Iba1, 019-19741, WAKO Pure Chemical Industries ltd, Japan), and doublecortin (1:500, polyclonal goat anti-DCX, Santa Cruz Biotechnology, USA). Sections were rinsed, incubated for 1 h with block solution and biotinylated secondary antibodies (1:1000, Jackson ImmunoResearch Laboratories Inc, USA), rinsed and incubated with avidin-biotin-peroxidase (10 µL/mL TBS of A and B, Vectastain Elite ABC Kit, Vector Laboratories, USA) for 1 h. Sections were rinsed and developed in 3,3′-diaminobenzidine (DAB, Saveen Werner AB, Sweden) diluted in TBS with H_2_O_2_ and NiCl_2,_ until sufficient color was noted. After rinsing in tap water, sections were mounted using 0.1 M phosphate buffer, pH 7.5, and dried overnight, then cover slipped with X-Tra-Kitt (Medite GmbH, Germany).

### NeuroTrace Fluorescent Nissl stain

Sections were rinsed in TBS and then washed in TBS with 0.1% Triton X-100 for 10 min. After further washing, sections were incubated with NeuroTrace^TM^ 500/525 Green Fluorescent Nissl stain for 20 min (N21480, ThermoFisher Scientific, USA). Following several rinsing steps in TBS, the sections were mounted and cover slipped with ProLong® Gold Antifade Reagent (ThermoFisher Scientific, USA).

### Stereological procedures

Cells were counted in every 12th section using systematic-random sampling (Stereoinvestigator, MicroBrightField, USA) and a Leica DM6000 B microscope (Leica Microsystems, Germany). Counting started on sections containing a clearly divided dorsal and ventral hippocampus (only the dorsal granule cell layer [GCL] was measured). Total volumes were calculated according to the Cavalieri principle (*V* = SA × *P* × *T*, where *V* is the total volume, SA is the sum of area measurements, *P* is the inverse of the sampling fraction, and *T* is the section thickness). The total number of cells was obtained by dividing the number of counted cells with the sampling fraction. Volumes for corpus callosum and thalamus were also measured in sections eligible for hippocampal quantifications.

NeuroTrace was used to quantify neurons in GCL (×40 objective). A 275 × 75-µm grid was randomly placed over the traced area and counting frames (25 × 25 µm) were placed within the grid. The total number of GCL neurons per animal was calculated by dividing the number of counted cells with the sampling fractions, i.e., fraction of sampling area/total traced area × series fraction (1/12) × optical dissector height/physical section thickness.

### Statistics

For statistical analyses, two-way ANOVA was used (drug and treatment as main effects), followed by a post hoc test (Sidak, corrected for multiple testing using GraphPad Prism 7.02). Weight was analyzed using three-way ANOVA (Stata). Statistical significance was considered if *P* < 0.05.
